# The 2024 Endocrine Society Guideline on Vitamin D: Comprehensive Summary and Critical Appraisal

**DOI:** 10.3390/nu18091472

**Published:** 2026-05-05

**Authors:** Stefan Pilz, Pawel Pludowski, Daniel Arian Kraus, Lisa Schmitt, Uwe Riedmann

**Affiliations:** 1 Department of Internal Medicine, Division of Endocrinology and Diabetology, Medical University of Graz, Auenbruggerplatz 15, 8036 Graz, Austria; 2 Department of Clinical Biochemistry, The Children’s Memorial Health Institute, 04-730 Warsaw, Poland; pludowski@yahoo.com

**Keywords:** Vitamin D, guideline, endocrine society, supplementation, prevention

## Abstract

Background/Objectives: An Endocrine Society Clinical Practice Guideline on vitamin D was published in 2024. Its main objective was the use of vitamin D to lower the risk of disease in individuals without established indications for vitamin D treatment or 25-hydroxavitamin D (25(OH)D) testing. The methodology followed the Grading of Recommendations, Assessment, Development, and Evaluation (GRADE) and the Evidence-to-Decision (EtD) framework. Evidence from randomized controlled trials (RCTs) retrieved by a systematic review was prioritized to inform this guideline. It was concluded that vitamin D supplementation reduces rickets and respiratory tract infections in children, mortality in individuals aged 75 years or older, pregnancy complications (outcomes), and progression of prediabetes to diabetes mellitus. Consequently, empiric vitamin D supplementation was recommended for individuals aged 1 to 18 years and ≥75 years, pregnant women, and individuals with prediabetes. Empiric vitamin D supplementation is defined as a vitamin D intake that exceeds the Dietary Reference Intakes (DRIs) and is implemented without 25(OH)D testing. Methods: This article provides a comprehensive guideline summary and critical appraisal based on a narrative review on scientific publications regarding that guideline. Results: Several publications discussed the 2024 Endocrine Society Clinical Practice Guideline on vitamin D. The main criticisms and discussion relate to unclear vitamin D dosages, guideline applicability to certain populations including controversy with previous vitamin D guidelines, and the implications of 25(OH)D testing. Conclusions: The 2024 Endocrine Society Clinical Practice Guideline on vitamin D followed a rigorous methodological approach with high quality standards but it leaves many open questions and uncertainties warranting clarification.

## 1. Introduction

The Endocrine Society Clinical Practice Guideline on vitamin D for the prevention of disease was published in 2024 [1]. The main objective was to provide clinical guidelines for the use of vitamin D to lower the risk of disease in individuals without established indications for vitamin D treatment or 25-hydroxyvitamin D (25(OH)D) testing [1]. This publication may have enormous significance for the role of vitamin D in public health worldwide. Several scientific publications have already discussed some issues related to the guideline and there were several scientific presentations and debates on this issue, such as at the 7th International Conference “Vitamin D—minimum, maximum, optimum,” organized under the auspices of the European Vitamin D Association (EVIDAS) in October 2025 in Warsaw, Poland [2,3,4,5,6,7]. A comprehensive guideline summary for clinicians and public health combined with a critical appraisal based on existing articles discussing the 2024 Endocrine Society guideline on vitamin D is currently not available. We aimed to address this gap with this article, which may help with the implementation of this guideline and give some guidance for the future perspective of vitamin D and its clinical applications.

In this narrative review, we start with a comprehensive summary of the key recommendations of the Endocrine Society guideline on vitamin D (referred to in this article as the “guideline” if not otherwise specified). Then, we briefly refer to scientific publications discussing this guideline. Our critical appraisal of the guideline is separated in the sections (a) uncertainties regarding doses, (b) target population, (c) applicability and implementation, (d) laboratory testing, (e) new scientific evidence and (f) outlook.

## 2. Summary of the 2024 Endocrine Society Guideline on Vitamin D

The methodology of the 2024 Endocrine Society guideline on vitamin D followed the Grading of Recommendations, Assessment, Development, and Evaluation (GRADE) and the Evidence-to-Decision (EtD) framework [1]. Evidence from randomized controlled trials (RCTs) retrieved by a systematic review was prioritized to inform this guideline [8]. If available RCTs were insufficient, large longitudinal observational cohort studies (>1000 participants) were permitted if they included appropriate comparison groups and outcomes [1]. Importantly, this guideline applies to individuals without established indications for vitamin D treatment and testing for 25(OH)D, which is the established parameter to assess vitamin D status. As there are guidelines for vitamin D supplementation to prevent nutritional rickets in the first year of life, the Endocrine Society guideline 2024 refers to these publications regarding this demographic [1,9,10]. However, rickets prevention is not only relevant in newborns, as a study in Turkey showed rickets peak incidences at 0–2 years and at 12 to 15 years [11].

The main recommendations of the Endocrine Society guideline 2024 are that “the panel suggests empiric vitamin D supplementation for children and adolescents aged 1 to 18 years old to prevent nutritional rickets and because of its potential to lower the risk of respiratory tract infections; for those aged 75 years and older because of its potential to lower the risk of mortality; for those who are pregnant because of its potential to lower the risk of preeclampsia, intra-uterine mortality, preterm birth, small-for-gestational-age birth, and neonatal mortality; and for those with high-risk prediabetes because of its potential to reduce progression to diabetes [1].” Empiric vitamin D supplementation was defined as a vitamin D intake that exceeds the Dietary Reference Intakes (DRIs) and is implemented without testing for 25(OH)D. For reference, the DRI for daily vitamin D supply by the Institute of Medicine in the US (now known as the National Academy of Medicine) is 600 international units (IU) for ages 1 to 74 years old (including pregnant and lactating women) and 800 IU for all individuals aged 75 years and older (divide by 40 to convert IU to µg of vitamin D) [12]. The guideline panel noted that the optimal doses for empiric vitamin D supplementation remain unknown and suggests, for non-pregnant individuals ≥50 years with indication for vitamin D treatment, daily, lower-dose vitamin D instead of nondaily, higher-dose vitamin D [1]. For each recommendation, the guideline indicated, as a technical remark, the vitamin D supplementation doses (range and estimated weighted averages per day) of the clinical trials that were retrieved by the respective systematic review, but did not further discuss the dosages or refer to them in specific recommendations (see Table 1 for guideline recommendations and technical remarks). For individuals without an indication for empiric vitamin D supplementation, as mentioned above, the guideline panel continues to support and endorse the DRI recommendations of the Institute of Medicine [12]. Interestingly, the guideline panel judged that healthy adults aged 50 to 74 years old could rationally choose to take vitamin D supplements if they are not expected to have adequate vitamin D status via sun exposure and do not reliably meet DRI of vitamin D from vitamin D-containing or fortified foods.

Regarding vitamin D testing, the panel suggests against routine 25(OH)D testing in all populations considered, including adults with dark complexion and those with obesity [1,13]. As the panel did not find clinical trial evidence for 25(OH)D thresholds tied to outcome-specific benefits, this guideline no longer endorses the target 25(OH)D concentration of 30 ng/mL (75 nmol/L) and the respective classifications of vitamin D sufficiency, insufficiency and deficiency, as suggested by the 2011 Endocrine Society guideline on vitamin D [14]. In a correction to the 2024 Endocrine Society guideline on vitamin D, it was emphasized that this 2024 guideline replaces the 2011 guideline, to avoid the impression that the older guideline still applies [1,14]. While the objective of the 2011 guideline was to provide guidelines to clinicians for the evaluation, treatment and prevention of vitamin D deficiency with an emphasis on the care of patients who are at risk for deficiency, the 2024 guideline addresses individuals without established indications for vitamin D treatment or 25(OH)D testing [1,14].

## 3. Scientific Publications Related to This Guideline

We performed a PubMed literature search on 3 March 2026, using the search terms “Endocrine Society” and “guideline” and “vitamin D”, and retrieved several articles that specifically discussed the 2024 Endocrine Society guideline on vitamin D. These publications, along with our own reference lists and debates at various scientific conferences such as at the 7th International Conference “Vitamin D—minimum, maximum, optimum,” were used as the literature basis for this article [1,2,3,4,5,6,8,13,14,15,16,17,18,19]. Though important, we initially refrained from conducting a pre-specified formal appraisal of this guideline by using established checklists, such as the AGREE II (Advancing Guideline Development, Reporting and Evaluation in Health Care II) framework [20,21,22,23,24,25,26]. Such an approach has a strong focus on general methodological issues but does not cover many specific aspects related to vitamin D that we wish to discuss [20,21,22,23,24,25,26]. Thus, in this article, we elaborate on certain issues of this guideline that we consider to be of high practical relevance for clinicians and everyone with interest in vitamin D, though this non-systematic approach is a limitation of this work. We aimed to be reasonable and balanced in our guideline appraisal, but we cannot exclude our own confirmation bias regarding our previous publications and opinions, though we partly addressed this by finally considering the AGREE II framework during the revision of this manuscript (see Supplementary Table S1 for our AGREE II appraisal). We largely refrain from discussions on selection of studies and their interpretation as the evidence informing this guideline, and refer to respective publications already touching on these topics [2,3,4,6,7]. We appreciate the work of the panel that drafted the Endocrine Society guideline and agree with many of their statements, but aim to critically appraise certain issues that may deserve attention and clarification in future guideline revisions.

## 4. Critical Appraisal of This Guideline

### 4.1. Uncertainties Regarding Doses

The guideline states that the optimal doses for empirical vitamin D supplementation are unknown [1]. For the clinical trials retrieved by the systematic review and that informed each population-specific recommendation for empiric vitamin D supplementation, the panel indicated the range and estimated weighted average daily vitamin D dose (see Table 1) [1]. Apart from this technical remark, no specific guidance is given on how this information on ranges and estimated weighted average daily vitamin D doses should inform clinical practice of vitamin D supplementation [1]. Though it appears obvious that these indicated dosages may be used as a guidance for vitamin D supplementation, the readership of this guideline remains uncertain regarding the central question, i.e., “how much vitamin D should be taken?” [1]. Regarding the recommendation for individuals aged 75 years and older and for those with prediabetes, the panel notes that, in many/some trials, participants remained on their routine vitamin D supplementation with up to 800 IU and 1000 IU of vitamin D per day, respectively. For example, in the VITamin D and OmegA-3 TriaL (VITAL), concurrent use of such vitamin D supplements (up to 800 IU daily) in addition to study medication was about 43% overall [27]. After 5 years, the outside use of vitamin D supplements greater than 800 IU per day in the vitamin D and placebo group was even 6.4% and 10.8%, respectively [27]. Such data, along with relatively high baseline 25(OH)D concentrations in participants of these trials and already existing vitamin D food fortification in some countries such as the US, appear to be of relevance for vitamin D dosage recommendations, but how to deal with these issues is not covered by the guideline. The only clear statement regarding dosages is that empiric vitamin D supplementation exceeds the DRI, i.e., 600 IU for individuals below the age of 75 years and 800 IU for older ones [12]. The upper dose limit of empiric vitamin D supplementation is unclear in the guideline, though someone might refer to the Institute of Medicine report that the Endocrine Society guideline endorses for all individuals, in particular those not covered by its recommendations [12]. In this report, the safe tolerable upper intake level of vitamin D for those aged 9 years and older, 4 to 8 years and 1 to 3 years is set at 4000, 3000, and 2500 IU per day, respectively [12]. For individuals aged 50 years and older, the guideline suggests daily lower-dose vitamin D instead of nondaily, higher-dose vitamin D [1]. The panel does not specify lower doses and higher doses but notes potential risks for fractures and falls with intermittent vitamin D doses exceeding 100,000 IU as a bolus and higher fall risk with dosing intervals greater than 12 weeks [1]. In this context, we would consider doses up to 2000 IU daily as “low doses” in adults, though this is merely an expert opinion based on general dosage ranges according to the guideline and safety considerations [27,28,29,30,31,32]. It is certainly challenging to release recommendations when the evidence is heterogeneous and limited, but just noting that the optimal doses for empirical vitamin D supplementation are unknown, though true, is probably unsatisfactory for many readers who seek for practical guidance from such a guideline. This missing guidance is of particular concern, as empirical vitamin D supplementation is suggested for large parts of the general population. As guidelines should not abstain from providing guidance even if the evidence is limited, we encourage the panel of the Endocrine Society guideline to provide more clarity for vitamin D dosage recommendations rather than leaving this decision up to others such as non-experts and the lay public. At least a dosage range for vitamin D supplementation with a clear upper limit would be important regarding safety issues. The authors of this article consider a daily vitamin D supplement dose of 2000 IU as an effective and safe dose that may be suitable for the prevention and treatment of vitamin D deficiency in most adults, but this is only an expert opinion that is subject to discussion [33]. Importantly, a daily vitamin D dose of 2000 IU is within the dosage range of clinical trials providing data for all recommendations regarding empiric vitamin D supplementation of this guideline (see Table 1).

### 4.2. Target Population

The panel notes that this guideline applies to individuals without established indications for vitamin D treatment or 25(OH)D testing [1]. Specifically, the guideline does not address individuals with underlying conditions that substantially alter vitamin D physiology, including various conditions associated with decreased absorption (e.g., short gut, gastric bypass, inflammatory bowel disease), increased catabolism/decreased activation (e.g., some medications), and increased renal losses (e.g., nephrotic syndrome) [1]. In addition, this guideline does not cover persons known to be at high risk of fractures and those with chronic kidney disease or established indications for 25(OH)D testing (e.g., hypocalcemia) [1]. The guideline does also not clarify whether and how those who present with low levels of 25(OH)D should be evaluated and/or treated [1]. The DRIs for vitamin D of the Institute of Medicine are not replaced by this guideline and continue to apply to all healthy persons [12]. The target users of this guideline are also not clearly defined.

As the 2024 Endocrine Society guideline for vitamin D replaces the respective 2011 guideline, this creates a significant gap in guidance regarding vitamin D for several specific populations covered by the “old” guideline from 2011 but not addressed in the guideline from 2024 [14]. For reference, the objective of the Endocrine Society guideline from 2011 was to provide guidance to clinicians for the evaluation, treatment and prevention of vitamin D deficiency with an emphasis on the care of patients who are at risk for vitamin D deficiency [14,34]. After the publication of the 2024 Endocrine Society guideline and the retraction of the 2011 Endocrine Society guideline on vitamin D, it remains unclear how to take care of individuals with, e.g., reduced 25(OH)D concentrations, or those conditions or diseases (e.g., malabsorption) not covered by the 2024 guideline (and also not covered by the Institute of Medicine report on vitamin D). Michael Holick, the first author of the 2011 Endocrine Society guideline on vitamin D, discussed this issue and suggested to adhere to the 2011 guideline for populations who would remain without specific recommendations according to the 2024 guideline [2,3]. Simple retraction of the 2011 guideline without giving any advice or guidance for several populations of importance with regard to vitamin D may likewise cause confusion and uncertainties among health-care professionals and affected individuals [14]. Clarification of this issue is required, possibly by referring to one or more of existing vitamin D guidelines and/or expert opinion papers on vitamin D [24,25,26,35]. We urge the guideline committee to provide official guidance to sources that address the populations who were covered in the 2011 guideline but not in the current guideline.

### 4.3. Applicability and Implementation

We wish to emphasize that the 2024 Endocrine Society guideline on vitamin D suggests empiric vitamin D supplementation for roughly half of the US population [1]. We consider this as a call for widespread vitamin D supplementation, though we did not have the impression that this message was appropriately delivered. For context, in the US, 43% of adults suffer from prediabetes according to data from February 2026, about 20% are aged 18 years and younger, almost 10% are older than 75 years and a small percentage of women in the reproductive age are pregnant [18]. Thus, even if we consider individuals with certain diseases or conditions for whom the 2024 guideline does not apply, almost half of the US population might remain with an indication for empirical vitamin D supplementation according to the Endocrine Society. At such a scale, guidance on implementation into clinical routine is needed, especially as this constitutes a quality criterion of a guideline [21]. There are several challenges on how to translate the guideline recommendations for widespread empiric vitamin D supplementation into daily practice. While it is straightforward to identify individuals based on age (i.e., 1 to 18 year old children and adolescents, and individuals aged 75 years and older) and pregnant women, prediabetes remains unrecognized in about half of the affected individuals [18]. For context, the American Diabetes Association (ADA) defines prediabetes in non-pregnant individuals if one of the following criteria is met: HbA1c 5.7–6.4% (39–47 mmol/mol) or fasting plasma glucose 100 mg/dL (5.6 mmol/L) to 125 mg/dL (6.9 mmol/L) (i.e., impaired fasting glucose (IFG)) or 2 h plasma glucose during a 75 g oral glucose tolerance test (OGTT) 140 mg/dL (7.8 mmol/L) to 199 mg/dL (11.0 mmol/L) (i.e., impaired glucose tolerance (IGT)) [36]. In the technical remarks, it is noted that the clinical trials informing this guideline recommendation regarding vitamin D primarily related to adults with high-risk prediabetes, identified as meeting 2 or 3 of the ADA criteria for prediabetes and those with IGT [1]. Each year, 5 to 10% of those with prediabetes progress to type 2 diabetes and it has been estimated that the number needed to treat with vitamin D for the prevention of diabetes mellitus is only about 30 [18]. Thus, it seems obvious that reinforcing measures to identify prediabetes persons and providing them information on empiric vitamin D supplementation would be a desirable action and we encourage diabetes associations worldwide to do so. In general, we suggest actions from public health authorities on empiric vitamin D supplementation for all the specific populations concerned by this guideline. This could include information campaigns and the adoption and inclusion of information on empiric vitamin D supplementation in general clinical guidelines for children, pregnant women, persons with prediabetes and individuals aged 75 years and older. In parallel, it would be ideal to assess whether and to what extent this guideline changes current practice. Such data on guideline implementation are critical to identify potential barriers for their implementation that may be improved by future guideline revisions and public health actions.

### 4.4. Laboratory Testing

In line with the US Preventive Service Task Force (USPSTF) and the “Choosing Wisely” campaign, the Endocrine Society guideline on vitamin D suggests against routine screening for 25(OH)D levels in healthy adults [37]. Specifically, they argue against routine screening for 25(OH)D in adults with obesity and adults with dark complexion. Though the guideline mentions that this recommendation relates to adults who do not otherwise have established indications for 25(OH)D testing (e.g., hypocalcemia), a clear and complete list of established indications for 25(OH)D testing is missing, as well as guidance on how to proceed with individuals in whom reduced 25(OH)D concentrations are detected. While the guideline panel stresses the insufficient evidence to support screening with 25(OH)D testing, they also acknowledge the “growing interest by patients and physicians in assessing vitamin D status”. The evidence supporting the recommendations of the guideline is well outlined in the guideline and an accompanying communication article, but a common real-life scenario is that individuals with indications for empiric vitamin D supplementation may ask for a 25(OH)D test before starting supplementation and for a follow-up measurement [1,38,39,40]. For example, one study from Switzerland indicated that 20% of the population were tested for 25(OH)D in 2018, but testing may have decreased to roughly 10% in 2022, which was mainly a consequence of a national coverage restriction [41,42]. It would seem logical that baseline and follow-up 25(OH)D levels, if available for any reason, may impact decisions regarding vitamin D doses. For example, if no advice is given by the guideline for individuals with measured 25(OH)D levels, a person with a high 25(OH)D concentration of concern or in the toxicity range may still start empiric vitamin D supplementation by following this guideline. Importantly, the guideline endorses the recommendations of the Institute of Medicine report on vitamin D that considers 25(OH)D concentrations above 125 nmol/L as levels that may be of concern [12]. It remains unclear whether the Endocrine Society guideline on vitamin D agrees with this concept. In this context, it should be considered that, when following the suggestions for empiric vitamin D supplementation, a certain proportion of individuals will achieve 25(OH)D concentrations above 125 nmol/L. This issue warrants clarification and consideration that, since the publication of the Institute of Medicine report on vitamin D over 15 years ago, many studies were published on the safety of vitamin D, potentially leading to reconsiderations of that threshold and the safe tolerable upper intake levels [28,29,32,43,44]. We propose an update on the safety of vitamin D supplementation by, e.g., following the framework of the Institute of Medicine report on vitamin D, including a clarification on the upper intake levels and the threshold of 25(OH)D (e.g., can it be increased to 150 nmol/L?) by the Endocrine Society guideline.

Regarding overall health outcomes, there is a wide consensus that no universal and clear threshold for 25(OH)D concentrations exists to indicate vitamin D supplementation and that no optimal 25(OH)D concentration can be clearly defined. There is also a wide consensus that a continuum exists for 25(OH)D concentrations and health outcomes ranging from deficiency to toxicity, though with a risk of various sorts of bias in the available literature. Being formally correct with statements on 25(OH)D testing based on the guideline methodology does not address practical challenges for individuals and clinicians having to proceed with data on 25(OH)D tests. Providing guidance for individuals with measured 25(OH)D concentrations to minimize the risk of vitamin D overdosing is, in our opinion, required by a Clinical Practice Guideline. Thus, there is an unmet need for practical recommendations that cover individuals with previous 25(OH)D tests, even if the current guideline does not recommend routine screening.

### 4.5. New Scientific Evidence

The panel stated that “this guideline will be reviewed annually to assess the state of evidence and determine if there were any developments that would warrant an update to the guideline”. We briefly discuss certain articles, mainly systematic reviews and meta-analysis on outcomes and populations for which empiric vitamin D supplementation is recommended, published after the systematic review of the Endocrine Society guideline (i.e., 28 December 2023) and deemed relevant by the authors of this article for this guideline [8].

A major systematic review and meta-analysis on vitamin D and acute respiratory tract infections by Jolliffe et al. was updated [45,46]. Though the point estimate for the overall effect of vitamin D supplementation on acute respiratory infections risk was similar to that in the previous publication with an updated odds ratio (OR) of 0.94 (95% CI 0.88 to 1.00), formal statistical significance was no longer achieved [46]. The OR (95% CI) in children aged 1 to 15 years remained significant with 0.74 (0.60 to 0.92), but meta-regression analysis did not suggest effect modification by age [46]. Several articles were published regarding the effects of vitamin D on diabetes risk and glucose metabolism that largely support the beneficial effect of vitamin D supplementation regarding these outcomes [17,18,47,48]. A major Cochrane review on vitamin D in pregnancy was also updated, and documented fewer statistically significant positive effects of vitamin D than the previous publication [49,50]. Notably, while the previous article included 30 studies, the updated paper removed 20 of these studies due to “awaiting classification” following assessments of trustworthiness, excluded one additional study and only added one new study [50]. The main findings of this update were that vitamin D may reduce the risk of severe postpartum hemorrhage and low birth weight [50]. As in the meta-analysis that informed the guideline, some neonatal and pregnancy outcomes, though statistically not significant, showed point estimates favoring the benefits of vitamin D [1,8,50]. Thus, these new data, in our opinion, do not question the recommendations for empirical vitamin D supplementation in pregnancy. Several other excellent articles on vitamin D and pregnancy were published but a reasonable summary of them would by far extend the scope of our work [51]. Regarding rickets and mortality, we did not identify major advances in the literature. In the systematic review and meta-analysis informing this guideline, all-cause mortality was reduced by vitamin D with high certainty and a RR (95% CI) of 0.96 (0.93–1.00) [8]. Interestingly, one RCT in 316 patients with clinically isolated syndrome typical for multiple sclerosis showed that 100,000 IU of vitamin D every 2 weeks over 24 months significantly reduced disease activity [52]. We are aware that more evidence is required for claims on vitamin D in multiple sclerosis treatment but want to stress that the dose of 100,000 IU vitamin D every 2 weeks was safe in that trial, providing additional encouraging safety data [52]. Overall, the mentioned publications, in our opinion, did not materially change the evidence that was used to inform the 2024 Endocrine Society guideline on vitamin D, and we are also not aware that the guideline panel considers a revision based on new evidence.

### 4.6. Outlook

Vitamin D deficiency remains a public health problem and requires efforts from the health authorities to improve the current situation, with large parts of the population having insufficient vitamin D supply [53,54,55,56]. After the publication of various large vitamin D RCTs over the last several years, it will likewise become challenging to acquire funding for further major trials in this field [57]. While we thus expect decreasing scientific publications on vitamin D in the future, we consider it as an urgent and imperative task to translate the existing knowledge on vitamin D, including the Endocrine Society guideline, into clinical practice. Education and implementation regarding vitamin D treatment should be of high priority, but is challenging due to a heterogeneous landscape of many different and partially conflicting guidelines and expert recommendations [23,24,25,26,58,59,60]. We suggest that publications such as the Endocrine Society guideline on vitamin D should be tied to measures regarding its uptake and efforts to maximize its implementation (we assume that the guideline panel works on this but still wish to stress this point). Beyond vitamin D supplementation, vitamin D food fortification, as already implemented in several countries such as the US, Canada or Finland, is a promising approach to reduce the burden of vitamin D deficiency [61,62]. We also suggest a more holistic approach to improve vitamin D status, including measures to reduce the prevalence of obesity, increase physical activity, and improve dietary habits, including a sufficient vitamin D intake by natural foods [15,63,64,65,66,67,68,69,70].

The elephant in the room though that is barely touched in vitamin D guidelines is ultraviolet-B (UV-B) or sunlight exposure [71,72]. Any approach to improve vitamin D status has to take into account that sunlight- or UV-B-induced vitamin D synthesis in the skin is usually the main source of vitamin D supply in the general population [71,72,73,74]. There are certainly harms and benefits associated with UV exposure [71,75]. Beyond its effect to increase dermal vitamin D synthesis, accumulating evidence indicates that UV exposure may also confer significant non-vitamin D-dependent health effects [71,76,77]. Recent observational studies suggest that the benefits of increasing UV or sun exposure may outweigh its adverse effects [77,78,79]. For example, several studies indicate improvements in overall survival or other major health outcomes in individuals with more versus less sun and UV exposure, though the evidence on this issue is not fully consistent [71,78,79,80,81]. The amount of sun exposure for optimal health is currently unknown and recommendations must consider several factors such as latitude, season, time of the day, skin type, sun exposed skin area, and sun protective behaviors [71,75,82,83]. There is no consensus on this issue, but common recommendations and estimates suggest a daily sun exposure time between late morning and afternoon (e.g., between 10 am and 4 pm) during warmer months in the range of 5 to 25 min for sufficient vitamin D synthesis [77,84,85]. Exploring the potential to improve vitamin D status and health outcomes by increasing natural (i.e., sunlight) and/or artificial (UV-emitting devices) UV exposure is thus an interesting and important topic for future research and potentially also for public health measures [71,86,87].

## 5. Conclusions

The 2024 Endocrine Society guideline on vitamin D is a critical contribution to guide the use of vitamin D supplementation in general populations worldwide. In this article, we critically appraised this guideline and discussed some uncertainties and barriers for translating the recommendations for empiric vitamin D supplementation in daily practice. We cautiously mention some suggestions for these issues and encourage efforts to implement the recommendations of the Endocrine Society guideline to improve vitamin D supply and health outcomes of all populations concerned.

## Figures and Tables

**Table 1:** Recommendations and technical remarks for empiric vitamin D supplementation by the 2024 Endocrine Society Clinical Practice Guideline on vitamin D.

Empiric Vitamin D Supplementation Is Defined as a Vitamin D Intake That Exceeds the Dietary Reference Intakes (i.e., 600 to 800 IU of Vitamin D Daily) and Is Implemented Without Testing for 25-Hydroxyvitamin D				
Population	Ages 1–18 Years	Ages ≥ 75 Years	Pregnancy	Prediabetes
Indication for empiric vitamin D supplementation	To prevent nutritional rickets and because of the potential to lower the risk of respiratory tract infections	Because of the potential to lower the risk of mortality	Because of the potential to lower the risk of preeclampsia, intra-uterine mortality, preterm birth, small- for- gestational -age birth and neonatal mortality	Because of the potential to lower the risk of progression to diabetes
Vitamin D estimated weighted averages in clinical trials	1200 IU per day	Approximately 900 IU per day	2500 IU per day	Approximately 3500 IU per day
Vitamin D dosage ranges in clinical trials	300 to 2000 IU daily	400 to 3333 IU daily	600 to 5000 IU daily	842 to 7543 IU daily
Additional technical remarks		Participants in many trials were allowed to remain on their routine supplements, including up to 800 IU of vitamin D daily		Participants in some trials were allowed to remain on their routine supplements, including up to 1000 IU of vitamin D daily

IU, international units.

## Data Availability

All data used for this article is publicly available.

## Supplementary Materials

The following supporting information can be downloaded at: https://www.mdpi.com/article/10.3390/nu18091472/s1, Table S1: AGREE II appraisal of the 2024 Endocrine Society Vitamin D Guideline with additional comments sections.

## Abbreviations

The following abbreviations are used in this manuscript:

25(OH)D	25-hydroxyvitamin D
RCT	Randomized controlled trial
IU	International units
OR	Odds ratio
IGT	Impaired glucose tolerance
CI	Confidence interval
UV	Ultraviolet
